# Scabies in 604 Patients: A Glimpse into the Disease Burden and Its Associated Mortality in Hong Kong

**DOI:** 10.3390/tropicalmed9100245

**Published:** 2024-10-19

**Authors:** Pascoe Ao Ting Lee, Samson Sai-Yin Wong, Kenneth Ho Leung Ng

**Affiliations:** Department of Clinical Pathology, Tuen Mun Hospital, Hospital Authority, Hong Kong, China

**Keywords:** bacteraemia, coinfection, community-acquired infections, *S* *arcoptes scabiei*, Scabies, *Staphylococcus aureus*

## Abstract

Scabies is a worldwide parasitic dermatosis with a significant health burden on the young and the elderly. Statistics about the prevalence of scabies in Hong Kong are not available. This is a retrospective study of patients from a regional hospital cluster in Hong Kong with microscopy-documented *Sarcoptes scabiei* infestations from January 2018 to December 2022. The condition was categorised into classical scabies and crusted scabies upon clinical presentation. Demographic data, comorbid diseases, mobility and residential status, seasonal variability, secondary bacterial infection, treatment and outcomes were described. These were compared between classic and crusted scabies. In total, 604 patients were identified, representing 51.65 per 100,000 discharged patients during the study period. The median age was 84 years and 54.5% were male. The majority (506 or 83.8%) came from residential care homes for the elderly. The mean time from admission to diagnosis was 8.8 days for community-acquired infestation. There were 564 and 40 cases of classic and crusted scabies, respectively. The two groups of patients were comparable in terms of residence in elderly homes, co-existing chronic illnesses, mobility, and time from admission to diagnosis. Forty-five (7.5%) patients had positive blood cultures temporally associated with scabies. Patients with crusted scabies were at higher risk for bacteraemia (7/40 versus 38/564, *p* = 0.022). Permethrin and benzyl benzoate were the most popular treatment regimens, with treatment failure observed in 59/397 (14.4%) and 18/173 (10.4%), respectively. There were 172 (28.5%) mortalities within 30 days of scabies diagnosis. Thus, the burden of scabies infestation is significant in Hong Kong. Hospitalised patients diagnosed with scabies are mainly senior citizens living in residential care homes for the elderly, suggesting reservoirs of *S. scabiei* in the community. Of concern, bacteraemic illnesses are common and significant mortality is temporarily associated with infestation. With a rising elderly population, there is a pressing need to understand and control scabies in Hong Kong. Our study did not find that common medical illness, besides immunosuppressive therapy, predisposed patients to crusted scabies. The crusted form of scabies was associated with a higher risk of bacteraemia. The current study provides a better perspective of the disease load of scabies in Hong Kong.

## 1. Introduction

Human scabies is caused by the mite *Sarcoptes scabiei* var. *hominis*, an ectoparasitic infestation [1] that preferentially affects children and the elderly, under-privileged sectors of the community, over-crowded populations, and tropical countries [2,3,4]. Transmission occurs via direct physical contact, usually over a prolonged period of time for new infections. The intense itch seen in scabies is due to a delayed hypersensitivity reaction and inflammation, which accounts for the relatively long incubation period of 2–6 weeks following primary infestation [4]. Although scabies is not life-threatening on its own, scabietic itch greatly degrades quality of life. Complications, most notably secondary bacterial infections, contribute to significant morbidity and sometimes mortality [2,4].

The two common clinical presentations are classical scabies and crusted scabies [4,5]. The typical case of classical scabies is notable for the fine, wavy and scaly lines (burrows) that end with a black papule (the parasite) that appear. Common areas of infection are the intertriginous areas, including the interdigital web spaces of hands, the subungual areas, wrists, axillae, abdomen, buttocks, and the breasts and genital areas of women and men, respectively [6]. Most affected individuals carry 10 to 12 mites at the time of diagnosis. In contrast, crusted scabies is characterised by an extremely high mite load with associated cutaneous reactions, often manifesting as psoriasiform and hyperkeratotic lesions, and is highly contagious. Patients who are immunocompromised are said to be at higher risk for crusted scabies. However, comparative studies are rare [7] and most supportive evidence comes from uncontrolled patient series [8] and case reports [5].

Diagnosis is usually clinically made in the outpatient setting when a pruritic cutaneous reaction is seen with the typical findings in classical scabies, often accompanied by an affected family member [5,9]. When patients experience little or no itch, when hyperkeratotic lesions predominate, or when there are co-morbid medical conditions, laboratory confirmation is required. This is achieved through microscopic examination of skin scrapings for the mites and/or their eggs or faecal pellets. First-line treatment for classical scabies is with a topical scabicide, and the standard agents recommended include permethrin and benzyl benzoate [5,10]. A combination of topical scabicide and oral ivermectin is indicated for crusted scabies [10].

Systematic studies on the severe complications and mortality associated with scabies are not available [2,5]. Secondary bacterial infection is a common complication associated with scabies [11]. Invasive disease associated with bacteraemia has been reported in case reports [12] but its frequency among affected patients is unknown. In a large global study on the impact of scabies, mortality was assumed to be zero [3]. This assumption is controversial [13] and mortality has been reported [12]. However, the negative impacts of a diagnosis of scabies on patient survival have not been studied.

Scabies has been recognised as a neglected disease by the World Health Organisation. Occurrences of scabies have been increasing across the world [14]. Recent studies on global statistics find the highest disease burden in Indonesia, China, and part of the Southeast Asia and Asia pacific countries [2]. Data from Hong Kong are distinctively lacking. The purpose of this study is to examine the scope of scabies and its potential impacts on individual and population health through a large cohort of scabies diagnosed in hospitalised patients.

## 2. Materials and Methods

This is a retrospective study conducted in the New Territories West Cluster (NTWC), a hospital cluster in Hong Kong with 4762 hospital beds that provides healthcare services to 15.8% of the population in Hong Kong [15]. NTWC consists of three acute hospitals (Pok Oi Hospital, Tin Shui Wai Hospital, and Tuen Mun Hospital), one psychiatric hospital (Castle Peak Hospital) and one hospital serving patients with severe intellectual disabilities (Siu Lam Hospital).

The study period was from January 2018 to December 2022. The diagnosis of scabies was made by collecting skin scrapings from patients and sending to the microbiology laboratory for microscopic examination. Patients admitted to any one of the five NTWC hospitals with microscopically documented *S. scabiei* were included. Clinical information and records were retrieved via the Clinical Data Analysis and Reporting System of the Hospital Authority. Patient demographics, residential background, other medical diagnoses, concurrent immunosuppressive therapy, anti-cancer chemotherapy and/or radiation therapy, and immunosuppression for solid organ transplant, occurrence of bacteraemia, the use of scabicidal treatment, treatment outcomes and mortality within 30 days of the scabies diagnosis were collected.

The cases were categorised into classic scabies and crusted scabies based on clinical description and/or photo-documentation. As the full effectiveness of scabicidal treatment usually takes up to two weeks, treatment failure was defined as a second positive finding of *S. scabiei* more than 14 days after the first microscopic diagnosis. Associated bacteraemia was defined as a positive blood culture collected and dated within 14 days before or after a positive microscopy result for *S. scabiei*. However, bacteraemia originated from non-cutaneous foci such as the urinary or respiratory tracts was excluded. Organisms that likely represented blood culture contaminants, for example, coagulase-negative staphylococci, *Bacillus* spp., and coryneform bacilli, were also excluded from the analysis if the clinical findings were not consistent with a genuine bloodstream infection.

Descriptive statistics were used in the study. For statistical analysis, categorical variables were compared using Fisher’s exact test and parametric variables were compared with Student’s t test. Statistical significance was defined by a *p*-value < 0.05.

## 3. Results

**Figure 1:**
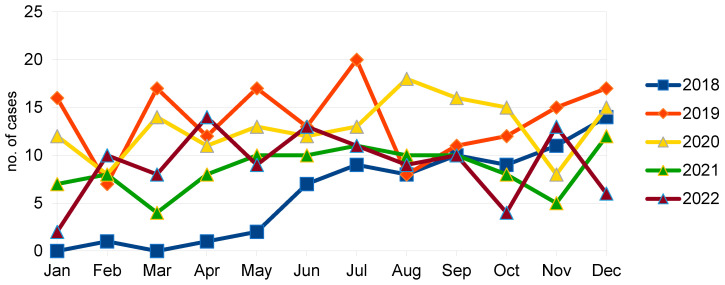
Statistics of the occurrence of scabies monthly comparison. Each year is represented by a separate line where each point corresponds to the number of laboratory diagnosis of scabies of the respective months. In other studies [17,18], seasonality was observed, but this is not apparent in our observation, possibly due to the relatively high humidity all year round [19].

Within the study period, 604 patients with microscopy-proven scabies were identified. With a total number of discharged patients (inclusive of deceased cases) of 1,169,426 during the same period [16], scabies was diagnosed in 51.65 per 100,000 patient encounters. The median age was 84 (range 22–108; interquartile range 74–90) years. A slight male predominance (329, 54.5%) was observed. The majority of the patients (506, 83.8%) came from residential care homes, and were unable to ambulate, being either chairbound (297, 49.1%) or bedbound (160, 26.5%). The most common comorbid conditions included hypertension (366, 60.6%), stroke (315, 52.2%), neurodegenerative disorders (280, 46.4%), diabetes mellitus (230, 38.1%), and heart diseases (140, 23.2%). Most of the patients were hospitalised under general internal medicine (490, 81.1%) (Table 1 and Table 2). The number of cases according to the month of diagnosis can be found in Figure 1.

With a reported incubation period of up to 8 weeks for scabies [17], the infections were presumed to be community-acquired when the diagnosis was made 56 days or less after admission. The majority of patients had community-acquired scabies (531, 87.9%). Seventy-three patients acquired the infection nosocomially.

Treatment records were available in 596 cases. Similar to the therapeutic experience reported previously in Hong Kong [20], permethrin was the most frequently prescribed topical scabicide (397, 66.6%), followed by benzyl benzoate (173, 29.0%). Treatment failure, defined by a positive finding of the parasite two weeks after treatment, occurred after permethrin alone in 57 (14.4%) and benzyl benzoate alone in 18 (10.4%) patients. The difference was not statistically significant (*p* = 0.14).

Forty patients (6.6%) were classified as having crusted scabies. They were comparable with patients with classic scabies in terms of residential background, ambulatory status, comorbid conditions, rapidity of diagnosis, and rates of treatment failure. The details are tabulated in Table 1. However, a higher proportion of patients with crusted scabies were immunosuppressed (10% vs. 1.4%, *p* = 0.0056).

There were 45 patients (7.5%) with concurrent bacteraemia temporally associated with scabies infestation (for the breakdown of the microorganisms of the bacteraemia, see Supplementary Material Table S1). Among the documented organisms, *Staphylococcus aureus* accounted for 35 episodes of bacteraemia (Table 1). Patients with crusted scabies, compared with classic scabies, were at higher risk for any kind of bacteraemia (*p* = 0.02) and in particular *S. aureus* bacteraemia (*p* = 0.005).

There were 172 (28.5%) deaths within 30 days of the diagnosis of scabies. The risk of mortality was not statistically different between patients with classical scabies and crusted scabies.

## 4. Discussion

Scabies is endemic in Hong Kong and was once remarked to account for 4% of attendances in a government dermatology clinic [20]. A recent, point-prevalence study from residential homes for the elderly reported two cases (0.05%) of scabies among 3857 residents [21]. However, during a targeted study from a single elderly home over a period of two months, 14 cases (5.8%) of scabies were diagnosed among 241 elderly residents [22]. The starkly different figures suggest that scabies in the elderly can be easily missed from a snapshot visit. Although an intense itchiness is often described as a manifestation of scabies in the textbook, half of the affected residents by Sun et al. did not complain of the symptom [23]. Our experience also indicates that scabies in the elderly population can be easily missed from a single medical encounter. Even among the patients admitted to the hospital, the mean time to diagnosis after admission was more than eight days.

In 2015, there were more than 73,000 (about 7% of the elderly population) occupants in Hong Kong who had been placed in residential care homes [19]. The elderly population in Hong Kong has been increasing since, with those aged 65 years or above having risen from 15.6% in 2015 to 10.5% by 22.4% in 2023 [24]. With a median age of 84 years, a high proportion of residential status in elderly homes, and the majority of patients being immobilised, the cohort of patients in this study most probably represented the senior citizens requiring residential care rather than the general community in Hong Kong. Our data are also indicative of the endemicity and a probable reservoir of scabies among the elderly population placed in residential care. Globally, the elderly population represents a significant portion of the disease pool [25], and outbreaks of scabies in elderly residential care homes occur frequently [26]. As comorbid conditions are common and can be more overwhelming, early and accurate recognition of scabies could be undermined. Since *S. scabiei* is highly contagious, the implications of this pool of infested elderly residents to the wider community deserve further investigation.

The prevalence of scabies has never been studied in Hong Kong. The current study represents the largest cohort of patients affected by scabies ever reported in Hong Kong to date. Among under-developed countries, the World Health Organization has noted prevalence rates of 5 to 50% among children [3], and though there is generally a decreasing trend, children continue to be the majority group affected [25]. However, a rising trend has been observed in high-income North America, with a percentage increase in the age-standardised rate of incidence of 0.48 (95% CI: 0.36–0.59) from 1990 to 2017 [25]. Cases are believed to be more sporadic in high-income countries and hospital data may provide a window through which the population disease burden can be glimpsed. In the United States, based on the National Inpatient Sample, scabies was diagnosed in 29.3 per 100,000 admissions [27]. Thus, our findings of scabies diagnosed in 51.65 per 100,000 patient encounters suggest a much higher burden of the scabies infestation in Hong Kong compared with the West.

Chronic skin disorders such as atopic dermatitis are well known to pave the way for secondary bacterial infection at the affected cutaneous areas. However, invasive infection in the form of bacteraemia is exceedingly rare [28]. In some parts of the world, a high rate of bacterial superinfection has been observed in patients with scabies [11]. In part, the physical barrier against bacterial infection is first jeopardised by the mite’s burrowed habitat [29]. In addition, serine protease inhibitors secreted by *S. scabiei* may further compromise the host’s immune response, in particular the complement system [30]. Animal models and studies have shown significant changes in skin microbiota when infested by *S. scabiei*, promoting the growth of pathogenic microbial taxa in the affected skin [31]. In human infestation, *S. aureus* and beta-haemolytic streptococci, especially *Streptococcus pyogenes*, are recognised common opportunistic pathogens [32,33].

Our data show that the adverse consequences of scabies infestation are more than skin deep. The 7.5% rate of invasive bacterial infection in the form of bacteraemic illness is worrisome. Patients with crusted scabies are at increased risk for potentially lethal complications. Though the causes of deaths in our patient series were attributed to underlying chronic illnesses, it is plausible that those secondary bacterial infections might have contributed to their demise. In our patient series, staphylococcal infections account for the great majority of the bloodstream organisms cultured. While bacteraemic illnesses can often be recognised and treated promptly in the hospital, their occurrence and implication in residential care homes for the elderly is unknown.

Crusted scabies represents a more severe form of the infestation and is prone to misdiagnosis due to the atypical clinical manifestations. A recent systematic review targeting the period 1998 to 2023 identified 204 publications describing a total 683 cases of crusted scabies [34]. The condition primarily affected adults with 21% of cases being individuals under 21 years of age. In addition, 10.2% of patients were overtly immunocompetent without any identifiable risk factors. Systemic immunosuppression from inherited, acquired, or iatrogenic causes was present in 495 (72.5%) cases. From another case series and literature review of crusted scabies from 2011 to 2021, 23 cases were tabled [35]. Most were adults (82.6%) and seven (30.4%) did not have any associated risk factors. With regard to immunosuppression, four patients (17.4%) had human immunodeficiency virus (HIV) infection and two (8.7%) were receiving immunosuppressive therapy after organ transplantation. Nonetheless, as these are case reports or case series of crusted scabies, the observations are heavily biased and it is not possible to tell if these are true predisposing factors. In contrast, Brites et al. were the first to compare patients with crusted scabies to classical scabies [8]. They studied 91 Brazilian patients with different forms of scabies and noted patients infected with human T-lymphotropic virus type 1 (HTLV-1) were at increased risk for crusted scabies compared with classical scabies (4/6 vs. 0/22, *p* = 0.0007). The association was more significant for patients co-infected with HTLV-1 and HIV (17/19 vs. 0/22, *p* < 0.00001). However, infection with HIV alone was not a risk factor (0/2 vs. 11/33, *p* = 1.0), even though it was singled out as one of the risk factors by Bergamin et al. [34] and Niode et al. [35]. Similarly, chronic medical illnesses were reported to be risk factors in these two reviews [34,35]. But from our study, chronic cardiovascular, neurological, psychiatric, dermatological disease and diabetes were equally distributed between patients with crusted scabies and classical scabies. The only significant predisposing factor for crusted scabies is immunosuppression as a result of systemic corticosteroid and anti-cancer therapies.

### Limitations

As only patients with laboratory-confirmed scabies were included, the study did not capture the full extent of the infestation among all the hospitalised patients. While we recognise this is not the only way to diagnose scabies [5,10], this approach could eliminate cases of dermatoses falsely diagnosed as scabies in those patients with concurrent dermatological illnesses. The number of hospital-acquired scabies was likely an underestimation, as used the maximum incubation period to exclude community-acquired infestations, and patients who had previously been sensitised to *S. scabiei* would become symptomatic much sooner, which had not been accounted for in our study. Even though we tried to eliminate cases of bacterial sepsis that were not of cutaneous origin, the retrospective nature of the study, with a reliance on the accuracy of medical records, could have over-estimated the relationship between scabies and bacteraemia. As the electronic health records only showed medication dispensing history but not the records detailing the exact times of medication administration, we were unable to ascertain the compliance to treatment recommendations. The mortality in this cohort of patients was all ascribed to concurrent medical illnesses. Prospective studies are required to examine the exact role of scabies in relation to patient demise.

## 5. Conclusions

The burden of scabies among hospitalised patients in Hong Kong is significant, with the majority of patients being elderly from residential care homes who may constitute a reservoir of *S. scabiei* in the community. Bacteraemic illnesses are common and mortality within 30 days of scabies diagnosis is high. With an ever-increasing elderly population, there is a pressing need to uncover the extent of scabies among the elderly community in residential care homes, control its spread, and treat it effectively to prevent fatal complications. Hospital staff, especially those working in high-income regions of the world, should familiarise themselves with the diagnosis and management of scabies. Crusted scabies, the type of scabies that carries a higher risk of bacteraemia, is poorly understood in aetiology and previously described risk factors are no longer relevant in comparative studies.

## Figures and Tables

**Table 1:** Residence, comorbidities and mobility of patients with classical and crusted scabies.

		Classical	Crusted	*p*-Value
Total		564	40	
Sex	Male	307 (54.4%)	22 (55.0%)	NS
Age	Mean (SD)	81.11 (12.29)	80.48 (13.38)	NS
Residence	Residential care homes	472 (83.7%)	34 (85.0%)	NS
Comorbidities	Diabetes mellitus	213 (37.8%)	17 (42.5%)	NS
	Hypertension	339 (60.1%)	27 (67.5%)	NS
	Stroke, intracranial haemorrhage, hypoxic brain injury	290 (51.4%)	25 (62.5%)	NS
	Neurodegenerative diseases, intellectual disability	263 (46.6%)	17 (42.5%)	NS
	Heart	134 (23.8%)	6 (15.0%)	NS
	Psychiatric conditions	53 (9.4%)	6 (15.0%)	NS
	Major orthopaedic conditions (e.g., hip fractures, vertebral injury)	114 (20.2%)	10 (25.0%)	NS
	Chronic skin conditions (e.g., eczema, bullous pemphigoid)	42 (7.5%)	3 (7.5%)	NS
Immunocompromised	Immunosuppressive therapy for solid organ transplant and autoimmune conditions; anti-cancer treatment	8 (1.4%)	4 (10%)	0.0056
Mobility	Patient data available	523 (92.7%)	36 (90.0%)	
	Mobile, aided or unaided (% of patient data available)	97 (18.5%)	5 (13.9%)	NS
	Chairbound/bedbound (% of patient data available)	426 (81.5%)	31 (86.1%)	
Seasonality	Warmer months (May to October)	307 (54.4%)	21 (52.5%)	NS
	Cooler months (November to April)	257 (45.6%)	19 (47.5%)	
Time to	≤56 days of admission	494 (87.6%)	37 (92.5%)	
Diagnosis	Mean (SD), days	8.77 (12.36)	8.81 (10.52)	NS
Persistence		75 (13.3%)	4 (10.0%)	NS
Bacteraemia	All organisms	38 (6.7%)	7 (17.5%)	0.0224
	*Staphylococcus aureus*	28 (5.0%)	7 (17.5%)	0.0055
Mortality	≤30 days from scabies diagnosis	159 (28.2%)	13 (32.5%)	NS

SD—standard deviation; NS—not significant.

**Table 2:** Distribution of patients’ medical subspecialties.

Category	Number (%)
Total	604
Emergency medicine	25 (4.1)
Intensive care and cardiac care units	8 (1.3)
Internal medicine	490 (81.1)
Surgery	38 (6.3)
Orthopaedics and traumatology	25 (4.1)
Neurosurgery	4 (0.7)
Psychiatry	9 (1.5)
Others (oncology, hospice and infirmary)	5 (0.8%)

## Data Availability

The original contributions presented in the study are included in the article, further inquiries can be directed to the corresponding author.

## Supplementary Materials

The following supporting information can be downloaded at: https://www.mdpi.com/article/10.3390/tropicalmed9100245/s1, Table S1: Microbiological profiles of scabies patients who had bacteraemia.
