# Supplementary material for: Scabies in 604 Patients: A Glimpse into the Disease Burden and Its Associated Mortality in Hong Kong
Source: Trop Med Infect Dis. 2024 Oct 19;9(10):245. doi: 10.3390/tropicalmed9100245 (PMC11511146; doi:10.3390/tropicalmed9100245)
Supplement: Supplementary file 1 [file tropicalmed-09-00245-s001.zip › tropicalmed-3253220-supplementary.pdf]

Supplementary material for: Scabies in 604 patients: a glimpse into the disease burden and its associated mortality in Hong Kong

Table S1. Microbiological profiles of scabies patients who had bacteraemia, classified by type of scabies, characteristic of bacteraemia, and listed in ascending order of age of the patients.

No.	Age (years)	Sex	Organisms
Classical scabies			
			Monomicrobial
1	63	F	MRSA
2	69	M	MRSA
3	71	M	MRSA
4	72	M	MRSA
5	73	M	MRSA
6	74	M	MRSA
7	76	M	MRSA
8	77	M	*Morganella morganii*
9	77	F	MRSA
10	78	M	SDSE
11	80	F	*Escherichia coli*
12	81	F	MRSA
13	81	M	MSSA
14	82	F	MRSA
15	82	M	MRSA
16	83	F	MRSA
17	84	M	MRSA
18	85	F	MRSA
19	86	M	*Escherichia coli*
20	86	F	*Klebsiella pneumoniae*
21	86	M	MRSA
22	86	M	MRSA
23	87	F	SDSE
24	88	M	MRSA
25	89	F	MRSA
26	89	F	MSSA
27	90	F	MRSA
28	91	F	MSSA
29	92	M	*Escherichia coli*

30	93	F	MSSA		
31	93	M	MSSA		
32	95	M	MRSA		
34	99	M	MSSA		
35	100	F	SDSE		
			Polymicrobial		
	Organisms		1	2	3
36	47	M	*Streptococcus agalactiae*	*Citrobacter freundii*	*Proteus mirabilis*
37	87	F	MRSA	SDSE	
38	91	M	SDSE	*Escherichia coli*	
33	97	F	MRSA	SDSE	
Crusted scabies					
			Monomicrobial		
39	62	F	MRSA		
40	70	F	MSSA		
41	79	M	MRSA		
42	86	F	MSSA		
43	87	M	MRSA		
44	90	F	MRSA		
45	94	F	MSSA		

M – male; F – female; MSSA – methicillin-susceptible *Staphylococcus aureus*; MRSA – methicillin-resistant *S. aureus*; SDSE – *Streptococcus dysgalactiae* subspecies *equisimilis*
All bacteraemia in patients with crusted scabies were monomicrobial.
